# Achievements of the COVID-19 Turkey Platform in vaccine and drug development with an approach of “co-creation and succeeding together”

**DOI:** 10.3906/sag-2112-178

**Published:** 2021-12-17

**Authors:** Hasan MANDAL

**Affiliations:** 1 The Scientific and Technological Research Council of Turkey, Ankara Turkey

**Keywords:** Vaccine, drug, COVID-19, SARS-CoV-2, co-creation

## Abstract

Mobilizing the research ecosystem for accelerating vaccine and drug development has been an important reality of the pandemic. This article reviews the scientific advances that are attained by the COVID-19 Turkey Platform for vaccine and drug development against the SARS-CoV-2 virus. The platform that is coordinated by the Scientific and Technological Research Council of Turkey is established with a “co-creation and succeeding together” approach, which involves 436 researchers across 49 different institutions working on 17 vaccine and drug development projects in total. Recent advances of the COVID-19 Turkey Platform include the fourth virus-like particle-based vaccine candidate in the world to enter clinical studies based on the World Health Organization COVID-19 vaccine tracker that is currently completing phase 2 clinical studies on the path towards initiating phase 3 clinical studies. Moreover, an adjuvanted inactivated vaccine candidate and two drug candidates that have been identified through the virtual scanning of more than 20,000 molecules are currently in clinical studies. Other vaccines and drug candidates involve additional innovative aspects, and a locally synthesized drug is found to have an impact on COVID-19. This review article discusses the advances that are achieved by the COVID-19 Turkey Platform from the ecosystem perspective, emphasizing the important scientific advances that have been achieved in the field of medical sciences.

## 1. Introduction

Mobilizing the ecosystem to strengthen the vaccine and drug development value chain against the COVID-19 pandemic has been imperative to have a positive impact on a healthy society. About one and a half months before the declaration of COVID-19 as a pandemic by the World Health Organization (WHO) [1], the first meeting that would lead to the development of vaccine and drug candidates was held in Turkey under the leadership of the Ministry of Industry and Technology [2]. This initiative led to the launch of the COVID-19 Turkey Platform for vaccine and drug development that is coordinated by the Scientific and Technological Research Council of Turkey (TUBITAK). A total of 436 researchers from 49 different institutions have taken part in the COVID-19 Turkey Platform, which has further expanded among professionals who have supported the production processes and clinical studies of the developed candidates, representing a great mobilization of the ecosystem.

**Figure 1:**
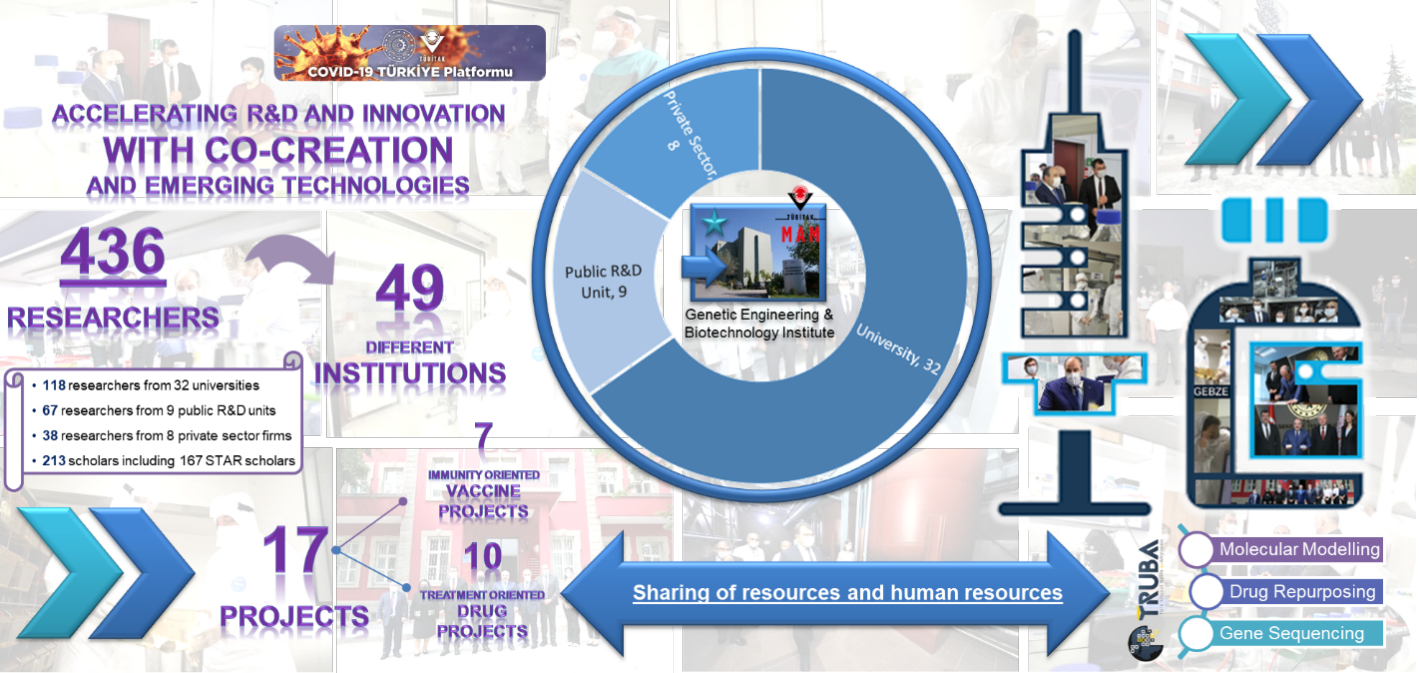
Representation of the co-creation based approach of the COVID-19 Turkey Platform.

Since its initiation, the COVID-19 Turkey Platform has been established based on the approach of “co-creating and succeeding together”, which has become an important approach for the achievement of the platform. The role of the COVID-19 Turkey Platform in mobilizing the ecosystem to achieve a positive impact based on vaccine and drug development against the pandemic was represented in earlier studies [3,4]. The unique platform has continued an intense and focused dedication to the sharing of resources, including human resources, as well as research infrastructure. This implementation has accelerated R&D processes with a focus on co-creation as a common approach. As summarized in Figure 1, the researchers who implemented the research projects have involved 118 researchers from higher education institutions, 67 researchers from public R&D institutes and units, 38 researchers from the private sector, and 213 young researchers, including 167 holders of STAR scholarships. The direct involvement of an important share of young researchers has supported the approach of the platform while ensuring its sustainability. The developments of the COVID-19 Turkey Platform were shared transparently in virtual conferences with public presentations [5,6]. There has been additional sharing of developments in an online portal, namely the COVID-19 Data Portal Turkey [7] that is associated with the European COVID-19 Data Portal [8], and periodic sharing of the most recent updates through public interviews. In addition to the responsibility of the COVID-19 Turkey Platform to the local ecosystem, the platform has broader international visibility with its being recognized as a model for co-creation based scientific approaches at the international level.

At the global level, an assessment, which was conducted eleven months after the rise of the SARS-CoV-2 virus, identified nearly 300 vaccine projects that were being focused in the scientific community, including new technologies [9]. The basic scientific approach in the SARS-CoV-2 virus characterization, including the structural glycoproteins and the domains of the Spike protein, has guided this process. At the same time, the overwhelming majority of vaccine candidates have focused on targeting the Spike protein. On the other hand, a more comprehensive vaccine design strategy can provide important advantages against certain instability challenges, especially during the mutation process.

**Figure 2:**
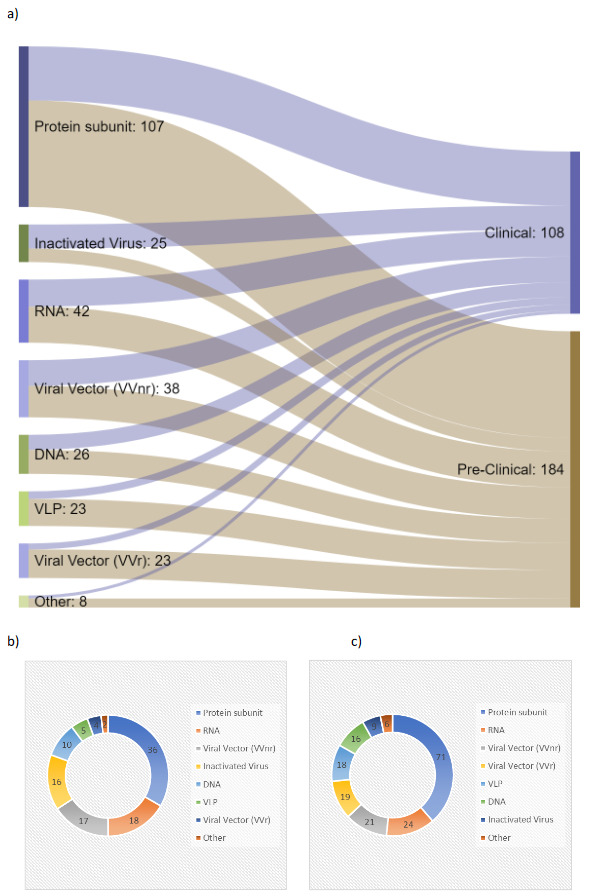
Distribution of vaccine candidates across types of vaccine technologies with distribution across the clinical and pre-clinical stages (a), distribution within clinical stages (b), and distribution within pre-clinical stages (c). Originally drawn based on [10] with July 27, 2021 data.

A recent update in the COVID-19 vaccine tracker and landscape of the WHO indicated a total of 292 vaccine candidates as of July 27, 2021 [10]. The Sankey diagram in Figure 2a indicates the flows among the different vaccine technologies based on the 108 vaccines in the clinical stages and 184 vaccine candidates in the pre-clinical stages as of July 27, 2021. As observed from the Sankey diagram in Figure 2a, the largest number of vaccine candidates is related to the protein subunit type, including recombinant Spike protein, which represents 33% of the total number of vaccine candidates in clinical studies and about 39% of the total number of vaccine candidates in pre-clinical studies. RNA based vaccine candidates, which is composed of 17% of the total number of vaccine candidates in clinical studies and 13% of the total vaccine candidates in pre-clinical stages, form the next category having the second largest number of vaccine candidates. Viral vector-based vaccines are reported based on the non-replicating (VVnr) and replicating (VVr) types in consistency with the data source [10]. As observed from Figure 2b, there are 5 virus-like particle (VLP) vaccine candidates in clinical studies, which represents about 5% of the total number of vaccine candidates in clinical studies. The distribution of vaccine candidates in pre-clinical studies are further represented in Figure 2c. In total, there are 23 VLP vaccine candidates either in clinical or pre-clinical studies in the world, which represents only 8% of the existing vaccine candidates in the scope of either stage.

## 2. Vaccine candidates of the COVID-19 Turkey Platform

A very important transformation was achieved with the approach of “co-creation and succeeding together” in the 7 vaccine development projects of the COVID-19 Turkey Platform. All of the different vaccine technologies that are being used across the world have been brought together in a single platform and brought to a higher level through adding new features to create innovative vaccine candidates of the COVID-19 Turkey Platform. This mobilization has strengthened the process of designing, developing, and producing innovative vaccine candidates under the strong pressure of the pandemic. Having been used to develop innovative vaccine candidates, these technologies of the COVID-19 Turkey Platform have been designed and developed to include important features in terms of efficacy.

The low percentage of VLP candidates in the total number of vaccine candidates against COVID-19 in the world as observed above in Figure 2 can be attributed to the fact that the development of this type of vaccine candidates can be technically challenging. In contrast to the technical challenges, VLP vaccine candidates can present important benefits, including good safety profiles, since the formulation does not include the genetic material of the pathogen [11]. Instead, the VLP vaccine candidates mimic the structure of the virus for the purpose of triggering a strong immune response.

The VLP vaccine candidate of the COVID-19 Turkey Platform mimics a non-infectious form of the SARS-CoV-2 virus. In addition, the design of this innovative vaccine candidate of the COVID-19 Turkey Platform targets four structural proteins based on the Spike protein as well as the Membrane, Nucleocapsid, and Envelope proteins (M, N, E). In this scope, it is recognized that the VLP technology has not been tested in any vaccine platform with the expression of these proteins together. The VLP vaccine candidate of the COVID-19 Turkey Platform is developed by design to provide immunization against the SARS-CoV-2 virus. The immunization range of the VLP vaccine candidate has also been increased by using an adjuvant that is licensed for safe for human use. Previously, a research team, including the scientists who developed this vaccine candidate had discovered that immune adjuvants can be improved through the inclusion of CpG. In particular, the use of synthetic oligodeoxynucleotides (ODNs) that contain unmethylated motifs of CpG was reported to promote the production of Th1 helper cells and pro-inflammatory cytokines, thereby increasing vaccine-induced immune responses [12]. Various studies had also reported the positive effect of CpG, including boosting immunity with antigen-specific responses [13] and improving the activity of vaccines with a good safety profile that target infectious diseases [14]. These important findings have been integrated into the VLP vaccine candidate as additional benefits beyond its targeting of the four structural proteins. 

With these innovative features, the VLP vaccine candidate of the COVID-19 Turkey Platform has become the fourth vaccine candidate in the world to enter clinical studies based on the WHO vaccine tracker within a few weeks after March 27, 2021. More specifically, the Phase 1 clinical study of the VLP vaccine candidate of the COVID-19 Turkey Platform has been undertaken and registered with identifier NCT04818281 [15]. The study involved 36 participants with randomized allocation and double-blind method that has been administered as two injections subcutaneously in a low dose at 10 µg (12 participants), a high dose at 40 µg (12 participants) as well as a placebo group. The Minister of Industry and Technology and the President of TUBITAK were volunteers in this first human clinical trials of the VLP vaccine candidate of the COVID-19 Turkey Platform [16]. The Phase 2 clinical trial of the VLP vaccine candidate of the COVID-19 Turkey Platform that initiated on June 26, 2021 has been conducted as a randomized, double blind, and multi-center clinical study across three different clinical centers in Ankara, İstanbul, and Kocaeli [17], and the efficacy, safety, and immunogenicity of the VLP vaccine have been assesed. This study included 330 participants and involved the original VLP vaccine that was developed for the Wuhan variant as well as another design that was developed against the Alpha (British) variant. Two groups, each composed of 110 participants, have received the first and second doses of either the original VLP vaccine or Alpha variant VLP vaccine versions. In addition, another 110 participants have been administered with one dose of each design 21 days apart. The clinical study involved volunteers aged between 18 and 59, and they were registered with the ID: NCT04962893 [18].

When compared to the information given in the most recent version of the WHO vaccine tracker, there is only one other VLP vaccine candidate that is in Phase 2/3 trials [19]. Two other VLP vaccine candidates are in Phase 1/2, and another is in Phase 1 clinical trials. The relevant clinical studies of VLP vaccine candidates are summarized in Table [10]. The developers are given in [10] with the VLP vaccine candidate in the first row as the VLP vaccine candidate of the COVID-19 Turkey Platform coordinated by TUBITAK. The Delta (Indian) variant of the SARS-CoV-2 virus will be also integrated in the Phase 3 studies of the VLP vaccine candidate of the COVID-19 Turkey Platform, which is a strong indicator of the strong research competency of the ecosystem. As of July 2021, the VLP vaccine candidate is completing the administration of second doses under Phase 2 studies while preparing for Phase 3 clinical trials that will also include the Delta variant. The candidate has the potential also to address international needs.

**Table:** Clinical study registrations of VLP vaccine candidates against COVID-19 [10].

VLP Vaccine Candidates	Phase 1	Phase 1/2	Phase 2	Phase 2/3
1	NCT04818281		NCT04962893	
2	NCT04450004		NCT04662697	NCT04636697
3		NCT04773665		
4		ACTRN12620000817943		
5	NCT04839146			

As another innovative candidate, the inactivated vaccine candidate of the COVID-19 Turkey Platform is developed to have advantageous qualities in terms of safety and efficacy. The advantageous features are obtained based on the contents that support the formulation of the inactivated vaccine candidate. As an advantage of working together in a collaborative platform with the approach of “co-creation and succeeding together”, it was observed that the highest antibody response is obtained when CpG is added to the aluminium hydroxide or Al(OH)_3_ adjuvant used in inactivated vaccines, which are the ones produced by the traditional vaccine technology. For this reason, an innovative feature of this candidate is the use of the Al(OH)_3_+CpG adjuvant within the formulation of the vaccine candidate. The Phase 1 clinical studies of the adjuvanted inactivated vaccine of the COVID-19 Turkey Platform are conducted with 50 participants who are aged between 18 and 45, and they were administered in two different doses. In the clinical study, 40 participants received doses that included 10 µg-3M inactivated virus or 20 µg-6M inactivated virus alongside 1 mg Al(OH)_3_ and 300 µg CpGODN adjuvanted vaccine, while 10 participants took place in the placebo group. After the administration of doses, which have been registered with identifier NCT04866069, in Phase 1 clinical studies [20], preparations towards the initiation of Phase 2 clinical studies of the inactivated vaccine are in the process of completion. 

Overall, the COVID-19 pandemic has accelerated the development of new types of vaccine platforms, including viral vector-based vaccines and mRNA vaccines [21] that are further represented in the COVID-19 Turkey Platform. The adenoviral vector vaccine candidate of the COVID-19 Turkey Platform is a viral vector-based vaccine that is designed to broadly recognize the antigens of the SARS-CoV-2 virus and to provide a strong and long-lasting immunity against them. It is also recognized that viral vector-based vaccine candidates can be successful vaccine technologies for creating a strong immune response to prevent the spread of the virus. In comparison to other adenoviral vector-based vaccines that are developed against this virus, the vaccine candidate of the COVID-19 Turkey Platform takes place as a second-generation vaccine that has a new design. Following the process of the development of VLP and adjuvanted inactivated vaccine candidates of the COVID-19 Turkey Platform, the adenoviral vector-based vaccine is planned to become the third vaccine candidate to initiate clinical studies.

Two other types of vaccine technologies provide an important level of flexibility for manipulating the coded antigen with rapid speed, namely the DNA and mRNA-based vaccines [9]. In the COVID-19 Turkey Platform, the DNA vaccine candidate is developed and is being produced at pilot scale based on Good Manufacturing Practice (GMP) for proceeding into clinical studies similar to the other vaccine candidates. Moreover, the mRNA based vaccine technology has gained a remarkable momentum during the COVID-19 pandemic [22]. The mRNA vaccine candidate that is being developed under the COVID-19 Turkey Platform is designed to be stable for 6 months at room temperature. The thermostable characteristic of the vaccine candidate is expected to provide an important advance. Moreover, recombinant viral protein technologies are known to provide protective efficacy; the effect of the recombinant vaccine against the hepatitis B virus infection is one of the major example regarding this efficacy [23]. Among the other vaccine technologies, the recombinant Spike protein vaccine candidate is being developed in the COVID-19 Turkey Platform with advances towards clinical studies. 

Another important innovation within the COVID-19 Turkey Platform is the development of the ASC particle technology based COVID-19 vaccine candidate. The apoptosis-associated speck-like (ASC) protein that is naturally present in human cells can spontaneously form particles when certain conditions are met. The formation of ASC particles is being used in the vaccine candidate of the COVID-19 Turkey Platform that is based on the ASC particle technology and is being developed as a novel vaccine technology in the world. Previously, processes that involved a supramolecular complex of the ASC speck were suggested to have an important role in cytosolic antigen presentation [24]. The new vaccine candidate has been progressing successfully towards GMP pilot production.

## 3. Drug candidates of the COVID-19 Turkey Platform

**Figure 3:**
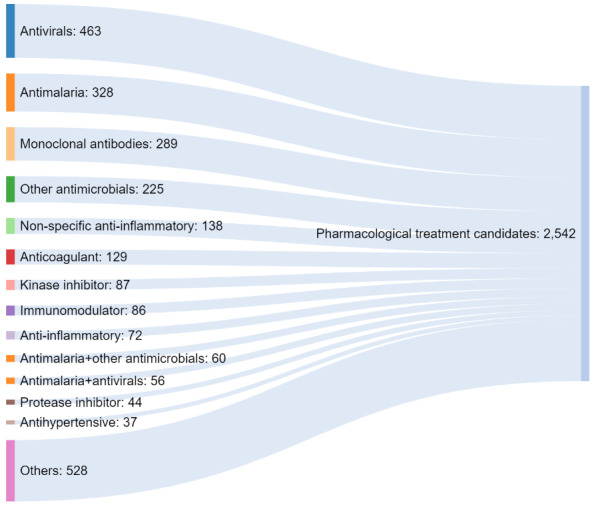
Distribution of different studies including pharmacological treatment candidates [26]. Originally drawn based on July 21, 2021 data.

Within the scope of the COVID-19 Turkey Platform, there are 10 different treatment-oriented drug development projects that involve local synthetic drug synthesis and production, drug molecular modeling, recombinant neutralizing antibody, and convalescent plasma related opportunities. Such a range of treatment-oriented drug development projects is compatible with the range of treatment opportunities that are being pursued in the world. In this scope, the COVID-NMA Initiative that includes the support of WHO aims to provide a dynamic mapping and systematic review of COVID-19 studies, including treatment-oriented drug development studies [25]. As of July 21, 2021, there were 2542 treatment oriented studies that were classified based on the type of pharmacological treatment in the COVID-NMA Initiative [26]. The distribution of these studies is represented in the Sankey diagram in Figure 3 with the largest share among the different types of pharmacological treatment being antivirals. Studies based on monoclonal antibodies are also among the top three types of pharmacological treatments as represented within the COVID-19 Turkey Platform. 

 Two of the antiviral based pharmacological treatment opportunities that are represented in this database are provided by the drug development projects of the COVID-19 Turkey Platform based on two different opportunities that have been identified based on the virtual scanning of over 20,000 molecules. One of these studies is based on the evaluation of the efficacy and safety of the use of Favipiravir and Ribavirin in the process of treating patients diagnosed with COVID-19 within 72 h [27]. As a guanosine analog that is able to interfere with the replication process of RNA and DNA viruses [27], the virtual screening had identified Ribavirin as a promising candidate that is represented in this clinical study. Previously, Ribavirin was also used during the outbreak of SARS in combination with other treatments that have an anti-inflammatory effect. Most recently, the studies of the research team of the COVID-19 Turkey Platform provided a detailed molecular analysis that showed its inhibitory effect on decreasing the TMPRSS2 enzyme [28]. The related clinical study is being undertaken with 100 participants with identifier of NCT04828564 [27].

Favipiravir that has been applied successfully for supporting the treatment of the COVID-19 infection in Turkey is a result of another important achievement of the COVID-19 Turkey Platform. The synthesis of the Favipiravir active ingredient involved a total of eight steps was synthesized in laboratory and industrial scale with completely domestic and national capabilities. In the project, different areas of expertise and production competencies, from organic chemistry synthesis to pharmaceutical technology, were brought together and a licensed pharmaceutical product was presented to the society through university-industry cooperation. In the first months of the pandemic on July 10, 2020, the bioequivalence study for the generic drug was completed, and its license was obtained. Since then, the locally synthesized and produced drug that contains the Favipiravir as active ingredient is being used successfully to support the treatment of COVID-19. The achievement has shown the importance of university-industry collaboration for positive impact. In addition to Favipiravir, the team has also locally synthesized Hydroxychloroquine and Remdesivir.

**Figure 4:**
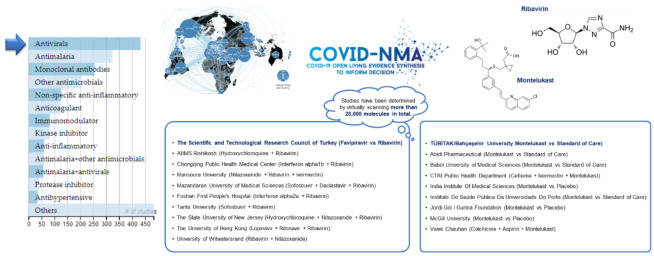
Representation of pharmacological studies with a focus on Ribavirin and Montelukast.

Based on a drug repurposing approach, another achievement of the COVID-19 Turkey Platform is the identification of the Montelukast molecule that is observed to prevent the SARS-CoV-2 virus from entering human cells and inhibit the main protease enzyme to avoid reproduction within the cell [29]. In this way, the agent is found to demonstrate a dual inhibition profile. The process of identification was based on virtually scanning over 15,000 molecules with a special algorithm, and then biological tests were applied to 25 molecules. Similar algorithms have been used previously to further identify multi-target HIV agents in other studies [30]. The project has benefited from a multidisciplinary approach for identifying opportunities in drug repurposing studies as well as the local competence of local drug synthesis. As a leukotriene (D4) receptor antagonist, Montelukast was identified as a novel agent that focuses on two important targets of the SARS-CoV-2 virus [31]. The Phase 2 study has been initiated with 380 participants that experimentally compares Montelukast, Montelukast plus Favipiravir and only Favipiravir as the standard treatment [31]. The two main studies that have reached the stage of clinical studies are further represented in Figure 4. 

Various compounds and lectins, such as Griffithsin, have been recognized to be possible inhibitors for coronaviruses in general, including the severe acute respiratory syndrome (SARS), Middle East respiratory syndrome and the avian infectious bronchitis virus [32]. Another achievement of the COVID-19 Turkey Platform has focused on the Griffithsin molecule in its natural form for preventing the infection that is caused by the SARS-CoV-2 virus. Important progress has been made for the utilization of the Griffithsin molecule for treatment against COVID-19 based on its properties for preventing and avoiding viral replication. Moreover, as proteins protect the human body against external threats such as viruses and bacteria, monoclonal antibodies have been identified as another promising opportunity for the treatment of COVID-19. Utilizing competences in biotechnological pharmaceutical drug development based on biosimilar drug production, existing infrastructure and technologies were mobilized for antibody-based drug therapy candidate against the SARS-CoV-2 virus for the treatment of COVID-19 patients. As another opportunity, Sarseptin has been developed as a drug candidate for temporary protection of high-risk groups that may not be vaccinated.

Among anti-inflammatory studies that are relatively less represented in the pharmacological treatment candidates in the world (Figure 3), another project of the COVID-19 Turkey Platform has focused on controlling and suppressing the inflammatory response. In particular, the Cytokine storm has been an important source of concern in the COVID-19 infection, since the severe inflammatory response damages critical organs, especially lungs and blood vessels. One project in the COVID-19 Turkey Platform has focused on the Interleukin-1 (IL-1) protein. While this protein provides natural defense and communication between immune system cells, it is also an important mediator of the cytokine inflammatory response. The project has focused on a stabilizing protein that may suppress increased levels of the IL-1 protein based on the IL-1 receptor antagonist (IL-1Ra, interleukin 1 receptor alpha) as an anti- IL-1 treatment opportunity. Certain results involving the use of anakinra were obtained, while it has not been the main driver of the antibody response [33].

In terms of the convalescent plasma therapy, the findings of the COVID-19 Turkey Platform indicate that it is more beneficial in the early stages of the disease, especially in people who are in the high risk group, rather than patients hospitalized in intensive care units. The dynamics of antibody levels and the antibody response in individuals who survived the COVID-19 infection were also observed.

## 4. Discussion of achievements in COVID-19 research 

**Figure 5:**
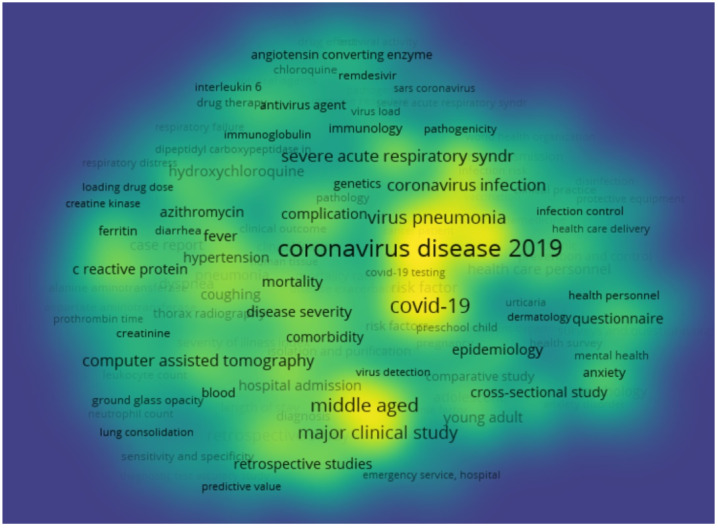
Mapping of the keyword occurrences of COVID-19 related publications in Turkey Originally drawn by using July 30, 2021 data.

In addition to the increase in scientific knowledge production in the COVID-19 Turkey Platform, the pandemic has spurred the scientific community around the world in better understanding the unknown mechanisms of the SARS-CoV-2 virus that cause the COVID-19 infection and its implications across medical sciences as well as society. Taking the most relevant key words that are most frequently observed together with COVID-19 and SARS-CoV-2, the scientific landscape has been mapped in the scope of the third Sustainable Development Goal (SDG) on “Good Health and Well-being.” These have included the key words of “coronavirus disease 2019”, “severe acute respiratory syndrome coronavirus 2”, and “SARS coronavirus”. The results of the mapping of the key words’ co-occurrences based on article and review publications in Scopus with authors from Turkey during the years 2020 and 2021 are summarized in Figure 5. In total, the mapping of the key word co-occurrences in the scope of SDG3 and COVID-19 related key words points out 1612 articles and review papers in total as of July 30, 2021. The color intensity in Figure 5 represents the co-occurrence frequencies.

Figure 5 represents a diverse contribution of scientific disciplines, including biochemistry, genetics and molecular biology, agricultural and biological sciences, chemical engineering, chemistry, computer science, decision sciences as well as arts and humanities. These scientific contributions have involved a focus on specific treatment opportunities, such as Hydroxychloroquine, Remdesivir and Chloroquine as well as broader keywords based on immunology and epidemiology. An interactive version is provided in Supplementary Material. Moreover, the source of 46 of publications with SDG3 and COVID-19 related key words is the *Turkish Journal of Medical Sciences* as the top journal of publication. The most cited five articles in this journal have focused on anti-inflammatory treatments [34], antiviral treatments [35], epidemiology [36], control measures [37], and pediatric patients [38]. These contributions are extended in the second special issue of the *Turkish Journal of Medical Sciences* that is dedicated to COVID-19.

The additional contributions in the second special issue of the *Turkish Journal of Medical Sciences* that has a focus on the first year of the COVID-19 pandemic updates include important evaluations, including those towards a new wave based on variants and booster vaccinations [39]. Other contributions have focused on policies during the pandemic and protective measures [40]. Additional contributions in recent issues of the *Turkish Journal of Medical Sciences* include a review of COVID-19 related scientific publications [41] as well as treatment of COVID-19 patients with Tocilizumab [42] and Favipiravir [43]. Clearly, the scientific community has been actively stimulated for COVID-19 research. In addition, TUBITAK has supported small and medium-sized enterprises in developing technological solutions, including innovative diagnostic kits and monitoring systems [44] and reference materials for RNA based diagnostic kits that were developed and commercialized in less than three months [45]. Moreover, recognizing that the pandemic has widespread impacts on society, TUBITAK supported 97 projects in the social sciences and humanities, including those addressing education, economics, business, sociology, communication, architecture, urban and regional planning, psychology, political science, and public administration. The policy relevant implications of the impact-oriented projects with a maximum duration of six months were shared in a virtual conference with 26 sessions [46] and published in a related booklet [47]. The role of the social sciences and humanities for solutions during the pandemic is highly relevant considering quadruple helix approaches that extends to society [48].

## 5. Conclusions for co-creation based approaches for impact

Advancing solutions against the pandemic has required an intense mobilization of entire ecosystems that includes the achievements of the COVID-19 Turkey Platform for vaccine and drug development. These achievements have taken place in an ecosystem where “co-creation and succeeding together” based approaches are being implemented intensively for impact-oriented solutions, including those that have an impact against the pandemic for better health and wellbeing. This review article has emphasized the achievements of the COVID-19 Turkey Platform based on this approach, including the advances regarding the fourth VLP vaccine candidate in the world on the path towards initiating its Phase 3 clinical studies. Moreover, the review article has placed the achievements on the sides of both vaccine and drug development in the broader context of progress at the global level based on the WHO vaccine tracker and COVID-NMA Initiative. The review article also provided a mapping of the scientific landscape of publications with SDG3 and COVID-19 related key words in Turkey, as the ecosystem is seeking to make a positive impact for a healthier society. 

The scope of this review article is to benefit a widespread understanding of these achievements while emphasizing the transformative role of the pandemic in conducting and implementing scientific advances based on a “co-creation and succeeding together” approach. Recently, opportunities for strengthening science systems in a post-COVID world are addressing similar advances [49]. These valuable experiences are highly relevant for accelerating progress towards sustainable development that is necessary for sustainability-oriented innovation systems [50] across multiple challenges, including but not limited to those in the field of medical sciences. Transformations are being diffused into opportunities for more sustainable and resilient societies [51], which is gaining importance every day for a healthy future of human civilization. 

## Supplementary Material

The file that provides an interactive version of Figure 5 is attached and can be viewed online [52].
